# Non-Mammalian Prdx6 Enzymes (Proteins with 1-Cys Prdx Mechanism) Display PLA_2_ Activity Similar to the Human Orthologue

**DOI:** 10.3390/antiox8030052

**Published:** 2019-03-01

**Authors:** Renata Bannitz-Fernandes, Rogério Aleixo-Silva, João Paulo Silva, Chandra Dodia, Jose Pablo Vazquez-Medina, Jian-Qin Tao, Aron Fisher, Luis Netto

**Affiliations:** 1 Departamento de Genética e Biologia Evolutiva, Instituto de Biociências, Universidade de São Paulo, São Paulo SP 05508-090, Brazil; bannitzfernandes@gmail.com (R.B.-F.); rlaleixosilva@gmail.com (R.A.-S.); silvajpn7@gmail.com (J.P.S.); 2 Department of Physiology, Institute for Environmental Medicine, Perelman School of Medicine, University of Pennsylvania, Philadelphia, PA 19104, USA; cdodia@pennmedicine.upenn.edu (C.D.); jpv-m@berkeley.edu (J.P.V.-M.); taoj@mail.med.upenn.edu (J.-Q.T.); abf@upenn.edu (A.F.); 3 Department of Integrative Biology, University of California, Berkeley, CA 94720, USA

**Keywords:** Peroxiredoxin, Prdx6, PLA_2_ activity, 1-Cys Prdx

## Abstract

Mammalian peroxiredoxin class 6 (Prdx6) are bifunctional enzymes. Non-mammalian Prdx6 enzymes display Cys-based peroxidase activity, but to date their putative phospholipase A_2_ (PLA_2_ activities) has not been experimentally investigated. Initially, we observed that five non-mammalian Prdx6 enzymes (enzymes from *Arabidopsis thaliana* (AtPER1), *Triticum aestivum* (TaPER1), *Pseudomonas aeruginosa* (PaLsfA) and *Aspergillus fumigatus* (AfPrx1 and AfPrxC)) present features compatible with PLA_2_ activities in mammalian Prdx6 by amino acid sequences alignment and tertiary structure modeling. Employing unilamellar liposomes with tracer amounts of [^3^H]-1,2-Dipalmitoyl-sn-glycero-3-phosphocholine (DPPC) and thin layer chromatography, all the tested non-mammalian Prdx6 enzymes displayed PLA_2_ activities, with values ranging from 3.4 to 6.1 nmol/min/mg protein. It was previously shown that Thr177 phosphorylation of human Prdx6 increases its PLA_2_ activity, especially at neutral pH. Therefore, we investigated if human Erk2 kinase could also phosphorylate homologous Thr residues in non-mammalian Prdx6 proteins. We observed phosphorylation of the conserved Thr in three out of the five non-mammalian Prdx enzymes by mass spectrometry. In the case of the mitochondrial Prdx6 from *A. fumigatus* (AfPrxC), we also observed phosphorylation by western blot, and as a consequence, the PLA_2_ activity was increased in acidic and neutral conditions by the human Erk2 kinase treatment. The possible physiological meanings of these PLA_2_ activities described open new fields for future research.

## 1. Introduction

Peroxiredoxins (Prdxs) are ubiquitous enzymes that play prominent roles in regulating peroxide levels within cells. These enzymes are often abundant and are capable of rapidly reducing peroxides [1]. Prdxs are Cys-based peroxidases that contain one or two conserved Cys residues [2]. To date, all characterized Prdxs display the universal motif PxxxT/SxxC and can reduce hydroperoxides efficiently, with rates ranging from 10^6^ to 10^8^ M^−1^ s^−1^ [3]. In spite of being a heterogeneous family, all Prdxs have this peroxidase activity dependent on Thr(Ser)-Cys-Arg catalytic triad that stabilizes the transition state of the reaction [4].

According to the PeroxiRedoxin classification index (PREX) database, Prdxs can be classified into six subfamilies: AhpC/Prx1, Prdx6, Prx5, Tpx, bacterioferritin comigratory protein (BCP)/PrxQ and AhpE, considering sequence and structural characteristics [5]. The Prdx6 subfamily is widespread from Eubacteria and Archaea to higher eukaryotes. It comprises almost exclusively 1-Cys Prdx mechanisms (only one Cys takes part in the catalytic cycle) and forms obligatory antiparallel dimers, and eventually, higher oligomeric states like decamers (pentamer of dimers). The peroxidase activity motif PVCTTE (Pro-Val-Cys-Thr-Thr-Glu) comprises the peroxidatic Cys (Cys_P_) and is characteristic of Prdx6 enzymes [6].

In addition to the peroxidase activity, mammalian Prdx6 displays a Ca^2+^-independent phospholipase A_2_ (aiPLA_2_) activity [7,8,9]. The PLA_2_ comprise a large family of lipolytic enzymes that hydrolyze the *sn*-2 acyl bond of phospholipids, releasing a free fatty acid and lysophospholipid [10]. The active sites of the peroxidase and aiPLA_2_ activities are spatially and functionally independent and the catalytic triad for human PLA_2_ activity is composed of the His26, Ser32 and Asp140 residues [11]. The identification of Ser32 residue in the GxSxG motif [7] as the site of phospholipid hydrolysis involved the use of serine protease inhibitors (e.g., MJ33, 1-hexadecyl-3-trifluoro-ethylglycero-s*n*-2-phosphomethanol), structural analyses and site-direct mutagenesis [12]. Afterwards, the lysosomal targeting signal peptide (GDSWGILFSHP) was determined and it also contains the catalytic Ser (Ser32) [13]. Notably, mutation of Ser32 to Thr abolished targeting human Prdx6 to lysosome-like structures while maintaining its PLA_2_ activity [14].

This aiPLA_2_ activity is maximum at pH 4, consistent with the subcellular localization of Prdx6 in a lysosome-like organelle. The phosphorylation of Prdx6 at Thr177 by MAP kinase induces a marked increase in PLA_2_ activity at acid and neutral pH, indicating that the PLA_2_ activity may also be important in the cytosolic environment [15].

The aiPLA_2_ activity is classically involved in pulmonary surfactant metabolism and membrane remodeling [16]. More recently, the Prdx6 PLA_2_ activity was also related to NADPH (Nicotinamide Adenine Dinucleotide Phosphate) oxidase 2 (NOX2) activation [17,18], and also with the metastasis and invasiveness of lung cancer cells [19].

So far, PLA_2_ activity has been identified only in mammalian Prdx6 enzymes. However, Prdx6 enzymes from other taxa also present structural similarities, suggesting that these enzymes might also possess PLA_2_ activity. Indeed, among 126 Prdx6 sequences analyzed in a previous study, 67% of the sequences had the lipase motif (38% had the classic lipase motif GxSxG, 11% had the alternative lipase motif GxSxA and 18% had variant lipase motifs) [6]. Therefore, we selected 5 non-mammalian Prdx6 enzymes to evaluate their putative PLA_2_ activity:

1. TaPER1 from *Triticum aestivum*: its gene is present as a single copy in the plant genome and is expressed mainly in the cytoplasm and nucleus during the development and germination of wheat seeds [20].
2. AtPER1 from *Arabidopsis thaliana*: its gene is also a single copy and its expression is induced by abscisic acid and oxidative stress [21]. AtPER1 also presents nuclear and cytoplasm localization [22]. Although knockout mutants for this gene are viable (unpublished data), overexpression or down-regulation of the AtPER1 gene affect seed germination during abiotic stresses [23].
3. AfPrx1 from *Aspergillus fumigatus* is a cytosolic protein that displays high reactivity for H2O2 (*k* = 2.28 × 10^7^ M^−1^ s^−1^) [24]. Moreover, the AfPrx1 is essential for the decomposition of exogenously added H2O2 as shown by the investigation of a knockout strain (Δaf*prx1*) [25]. The strain Δaf*prx1* is also more sensitive to other stressors’ molecules. Additionally, AfPrx1 plays a role in fungus virulence in neutropenic murine models [24].
4. AfPrxC from *Aspergillus fumigatus* is a mitochondrial protein that also reacts rapidly with H2O2 (*k* = 2.28 × 10^7^ M^−1^ s^−1^) [24].
5. PaLsfA from *Pseudomonas aeruginosa*: PaLsfA is the only 1-Cys Prdx among several antioxidant proteins in *P. aeruginosa*. PaLsfA expression is induced upon sulfate depletion [26] and it is involved with *P. aeruginosa* virulence [27].

## 2. Materials and Methods

### 2.1. Sequence and Structural Analysis

All the Prdx6 sequences studied here were obtained from the National Center for Biotechnology Information (NCBI) (https://www.ncbi.nlm.nih.gov/) and confirmed in other curated databases: Phytozome (https://phytozome.jgi.doe.gov) for TaPER1 and AtPER1; AspGD (http://www.aspgd.org) for AfPrx1 and AfPrxC; and Pseudomonas Genome DB (http://www.pseudomonas.com) for PaLsfA. The alignment was performed using Clustal Omega program (https://www.ebi.ac.uk/Tools/msa/clustalo/) [28], and the software Jalview (University of Dundee, Scotland, UK) [29] was used for visualization.

The structures of non-mammalian Prdx6 were modeled using the human Prdx6 (1PRX) as the template and the SWISS-MODEL tool (https://swissmodel.expasy.org/) [30]. The UCSF (University of California, San Francisco) Chimera program [31] was used to visualize and generate the images. The web based application WebLogo version 2.8.2 (http://weblogo.berkeley.edu, University of California, Berkeley, USA) was used to generate the lysosomal target sequence logo [32].

### 2.2. Cloning, Expression and Purification of Recombinant Proteins

The genes of the corresponding Prdxs were cloned in expression vectors as follows: pET16b/ta*per1* (kindly provided by Javier Cejudo) and pET15b/at*per1*; pET15b/pa*lsfA*. To clone into pET expression vectors (Novagen, Madison, USA), the sequences were amplified by polymerase chain reaction (PCR), from TOPO™ vector (Invitrogen, Carlsbad, USA) containing each sequence, using the following primers: TaPER1: 5′-AT**CATATG**CCGGGCCTCACCATCGG-3′ and 5′-AT**CTCGAG**CTAGACCTTGGTGAAGCG-3′, which contained sites for *Nde*I and *Xho*I restriction enzymes (bold letters). AtPER1: 5′-AT**CATATG**CCAGGGATCACACTAGG-3′ and 5′-AT**GGATCC**TCAAGAGACCTCTGTGTGACG-3′, which contained sites for *Nde*I and *Bam*HI restriction enzymes (bold letters). PaLsfA: 5′-CGCAT**CATATG**CTCAGACTCGGCGAC-3′ and 5′-AC**GGATCC**TCAGCGGTTGGGCTG-3′, which contained sites for *Nde*I and *Bam*HI restriction enzymes (bold letters).

The genes af*prx1* and af*prxC* were codon optimized for *Escherichia coli* expression and were synthesized by GenScript^®^ (Piscataway, USA). These genes were then cloned into the *Nde*I (5’) and *Bam*HI (3’) restriction sites of the pET15b plasmid. All constructions were confirmed by sequencing, using T7 promoter and terminator oligonucleotides (BigDyeTM Terminator v3.1 Cycle Sequencing Kit, Applied Biosystems®, Foster City, USA).

#### 2.2.1. Expression Procedures

Single colonies of *E*. *coli* BL21 (*DE3*) harboring the respective plasmid (pET16b/ta*per1*, pET15b/at*per1*, pET15b/af*prx1*, pET15b/af*prxC*, pET15b/pa*lsfA*) were cultured in Lysogeny broth medium (LB) containing 100 μg/mL ampicillin and grown for 16 h (37 °C) in an orbital shaker (250 rpm). The cultures were then diluted in 1 L of LB (100 μg/mL ampicillin) and were incubated at 37 °C until the optical density OD_600nm_ attained 0.6–0.8 values. Isopropyl β-D-1-thiogalactopyranoside (IPTG) was then added at a final concentration of 0.3 mM for AfPrx1, AfPrxC and 1 mM for AtPER1 and TaPER1. Then, each culture was incubated at 37 °C for an additional 3 h (250 rpm), with the exception of PaLsfA, in which 1 mM IPTG was added and the culture incubated at 30 °C for 6 h (250 rpm). Cells were harvested by centrifugation, and the pellet was washed and resuspended in the start buffer (20 mM sodium phosphate pH 7.4; 500 mM NaCl; 20 mM imidazole; 625 μM phenylmethylsulfonyl fluoride (PMSF). Twenty cycles of 15 s of sonication (30% amplitude) following 40 s in ice were applied to cell suspensions. Cell extracts were kept on ice during treatment with 1% streptomycin sulfate for 15 min and the suspension was centrifuged at 15,000 rpm for 45 min to remove nucleic acid precipitates and the insoluble fraction.

#### 2.2.2. Purification

Cell extracts were filtered and purified by immobilized metal ion affinity chromatography (IMAC) using the HisTrap™ FF 5 mL column (GE Healthcare, Chicago, USA), using the ÄKTAFPLC system (GE Healthcare, Chicago, USA). The conditions of protein purification were optimized using the gradient procedure for imidazole concentration as described by the manufacturer. Imidazole was removed from purified proteins by gel filtration using a PD10 column (GE Healthcare, Chicago, US). Purified proteins were stored in the following buffers: AtPER1 and TaPER1 in 20 mM Tris HCl buffer (pH 7.4) containing 500 mM NaCl, AfPrx1 and AfPrxC in 20 mM sodium phosphate buffer (pH 7.4) containing 500 mM NaCl, and PaLsfA in 20 mM HEPES buffer (pH 7.4) containing 150 mM NaCl and 0.1 mM diethylenetriaminepentaacetic acid (DTPA). Recombinant protein concentrations were determined spectrophotometrically at 280 nm. The extinction coefficients for reduced AtPER1 (ε_280nm_ = 24,013 M^−1^ cm^−1^), TaPER1 (ε_280nm_ = 21,555 M^−1^ cm^−1^), AfPrx1 (ε_280nm_ = 20,970 M^−1^ cm^−1^), AfPrxC (ε_280nm_ = 22,460 M^−1^ cm^−1^) and PaLsfA (ε_280nm_ = 33,920 M^−1^ cm^−1^) were determined using the ProtParam tool (http://www.expasy.ch/tools/protparam.html, Lausanne, Switzerland).

### 2.3. Preparation of Unilamellar Liposomes

The lipids used to make the unilamellar liposomes were: 1,2-Dipalmitoyl-sn-glycero-3-phosphocholine (DPPC) (Sigma-Aldrich P0763, Taufkirchen, Germany); L-α-Phosphatidylcholine (PC) (Sigma-Aldrich P2772, Taufkirchen, Germany); L-α-Phosphatidyl-DL-glycerol ammonium salt (PG) (Sigma-Aldrich P0514, Taufkirchen, Germany); cholesterol (Sigma-Aldrich C8667, Taufkirchen, Germany). The stock solutions were made by diluting the lipids in chloroform.

Unilamellar liposomes were made using DPPC/PC/PG/cholesterol (5:2.5:1:1.5) with tracer amounts of [^3^H] DPPC (American Radiolabeled Chemicals ART 0532, Saint Louis, USA). The lipid solution was evaporated to dryness under N_2_ and resuspended in isotonic saline. In some cases, the PLA_2_ inhibitor MJ33 was added (200 μM). The solution was repeatedly frozen/thawed by alternating liquid N_2_ and warm H_2_O (50 °C) (3 times), and then extruded at 50 °C for 10 cycles through a 200 μm pore size membrane to generate unilamellar liposomes using LIPEX™ (Transferra Nanosciences Inc., Burnaby, Canada). The liposomes were freshly prepared for every use and stored for short times at 4 °C [16].

### 2.4. Evaluation of PLA_2_ Activity

The PLA_2_ activities were determined using unilamellar liposomes (100 μL), containing tracer amounts of [^3^H] DPPC. Reaction mixtures contained non-mammalian Prdx6 enzymes (5, 10 or 50 μM) in an acid (40 mM sodium acetate pH 4, 5 mM ethylenediaminetetraacetic acid (EDTA)) or neutral pH buffer (50 mM Tris-Cl pH 7.4, 1 mM ethylene glycol-bis(2-aminoethylether)-*N*,*N*,*N*′,*N*′-tetraacetic acid (EGTA)), both incubated for 1 h at 37 °C. The reactions were interrupted by the addition of 3.75 mL of chloroform/methanol (1:2) for at least 1 h. The lipid extraction was performed by incubating samples with 1.25 mL of chloroform for 1 h, followed by addition of 1.25 mL of water and incubation for 1 h. The extracts were centrifuged for 10 min at 1000 rpm. The organic phase was separated and completely dried under N_2_ flow.

Thin layer chromatography (TLC) was performed in two steps with hexane/diethyl ether/acetic acid (1° step 60:40:10 and 2° step 90:10:1). After the run and plate drying, samples were stained in an iodine atmosphere chamber. The samples of interest were scrapped from the plate. The free fatty acid spot was quantified after extraction, as follows: 1 mL of water, 12 h of repose, addition of 10 mL of Econo-Safe™ Biodegradable Counting Cocktail (Research Products International, Mount Prospect, USA) and scintillation reading. The PLA_2_ activities were determined in counts per minute (cpm) after 1 h of incubation (subtracting the zero time from control) [16]. Cpm were converted to DPPC mass based on the specific activities of the starting material and were expressed as nmol of DPPC hydrolyzed/min/mg protein. Protein contents were measured by Coomassie blue assay, using the Quick Start Bradford Dye reagent (BioRad, Hercules, USA).

### 2.5. Phosphorylation Treatments of Non-Mammalian Prdx6

The human kinase Erk2 (GI: 119554) was selected for the in vitro phosphorylation assays (Merck 14-550, Darmstadt, Germany). Each one of the non-mammalian Prdx6 (150 ng/μL) was incubated in reaction buffer (50 mM Tris-Cl pH 7.5, 20 μM EGTA) containing 10 mM MgCl_2_ and 2 mM ATP in the presence or absence (as control) of active Erk2 kinase (10 ng/μL). The reactions were incubated at 30 °C for 90 min with slight agitation [33] and then separated for mass spectrometry (MS) analysis or for western blot assay as described below. The Erk2 kinase solution was previously diluted to 80 ng/μL, following the manufacturer instructions (50 mM Tris-Cl pH 7.5, 0.1 mM EGTA, with or without 0.1 mM Na_3_VO_4_, 0.1% 2-mercaptoethanol and 1 mg/mL bovine serum albumin (BSA)).

### 2.6. Reduction, Alkylation and Tryptic Digestion for MS

Samples obtained from the phosphorylation assay (described above) were mixed with 100 mM ammonium bicarbonate buffer pH 8 plus 8 M urea (1:1). Sequentially, samples were reduced with 10 µL of 10 mM dithiothreitol (DTT) for 1 h at 30 °C and were alkylated in the dark in an Eppendorf ThermoMixer (Eppendorf, Hamburg, Germany) by adding 10 µL of 500 mM iodoacetamide for 30 min at 25 °C (final volume 140 μL). After incubation, samples were diluted with 540 µL of 100 mM ammonium bicarbonate buffer pH 8. Finally, protein digestion was started by adding 3 µL of Trypsin Gold (Promega, Madison, USA) 40 ng/µL (protein/enzyme ratio of 50:1) for 16 h at 37 °C. The reaction mixtures were neutralized with 0.1% trifluoroacetic acid and were completely dried in speed vac. For purification, extracts were resuspended in 0.1% formic acid solution. Each sample was then concentrated through ZipTip resin (Merck, Darmstadt, Germany) according to the manufacturer’s protocol. Subsequently, samples were dried in speed vac and stored at −20 °C until use.

### 2.7. Evaluation of Thr Phosphorylation by MS

The resulting peptides were analyzed by liquid chromatography-tandem mass spectrometry (LC-MS/MS) using a nanoACQUITY UPLC system (Waters Corporation, Milford, USA) coupled to a TripleTOF 6600 mass spectrometer (AB SCIEX, Framingham, USA). Analysis was conducted under trap and eluted mode using a nanoACQUITY UPLC-Symmetry (Waters Corporation, Milford, USA) containing a C18 trap column (20 mm × 180 µm; 5 µm) and a separation column (75 µm × 150 mm; 3.5 µm). Trapping was done at 10 µL/min with 2% of solvent B. Peptides were separated with mobile phase A (0.1% formic acid in water) and B (0.1% formic acid in acetonitrile) at a flow rate of 0.4 µL/min using the following gradient: 2–35% B from 0 to 60 min; 35–85% B from 60 to 61 min; isocratic elution with 85% B from 61 to 65 min; 85–2% B from 65 to 66 min; isocratic elution with 2% B from 66 to 85 min. Nano-electrospray ion source was operated at 2.2 kV (ion spray voltage floating, ISVF), curtain gas 20, interface heater (IHT) 120, ion source gas 1 (GS1) 3, ion source gas 2 (GS2) zero, declustering potential (DP) 80 V. Time-of-flight mass spectrometry (TOF-MS) and mass spectrometry analyzis (MS/MS) data were acquired using information-dependent acquisition (IDA) mode. For IDA parameters, a 250 ms survey scan in the m/z (mass-to-charge ratio) range of 300–2000 was followed by 25 MS/MS ions in the m/z range of 100–2000 acquired with an accumulation time of 100 ms (total cycle time 2.8 s). Switch criteria included, intensity greater than 150 counts and charge state 2–5. Former target ions were excluded for 4 s. Software used for acquisition and data processing were Analyst® TF 1.7 (AB SCIEX, Framingham, USA) and PeakView® 2.2 (AB SCIEX, Framingham, USA), respectively. For the analysis of protein modification, MASCOT 2.4 software (Matrix Science Ltd., London, United Kingdom, Redoxoma-FAPESP user license 10.10.1.46/Mascot) was used with mass tolerance of 10 ppm for MS experiments and 0.05 Da for MS/MS experiments.

### 2.8. Evaluation of Prdx Phosphorylation by Western Blot

After the phosphorylation reaction, samples (10 μL) were reduced using DTT (100 mM), then heated (95 °C) for 5 min and analysed by SDS-PAGE (12%) (50 min/200 V). The gel was transferred to nitrocellulose membranes using NuPAGE® transfer buffer (Thermo Fisher Scientific NP0006, Waltham, US) (90 min/35 V). Ponceau S staining was used to verify the total protein amount. The blocking step was performed with buffer Odyssey (Li-Cor Biosciences 927-40000, Nebraska, USA) at room temperature for 90 min. The membrane was incubated with anti-phosphorylated Prdx6 primary antibody (anti-mouse P-Prdx6 S3091-1, Covance Research Products, Denver, USA) [17], diluted 1:200 in a mix with block buffer: TBS-T (1:4), overnight at 4 °C. TBS-T was used to wash the membrane (3 × 10 min). The incubation with secondary antibody anti-rabbit IRDye^®^ 800CW (Li-Cor Biosciences 925-32211, Nebraska, USA), diluted 1:5000 in TBS-T, was performed for 45 min at room temperature. The antibody to phosphorylated Prdx6 was generated by Proteintech (Chicago, USA) using a phosphorylated mouse Prdx6 peptide (TGTKPVApTPVDWKKG) that contains the Thr 177 and has been described and validated previously [17].

## 3. Results

### 3.1. Conservation of PLA_2_ Catalytic Triad in Non-Mammalian Prdx6

**Figure 1:**
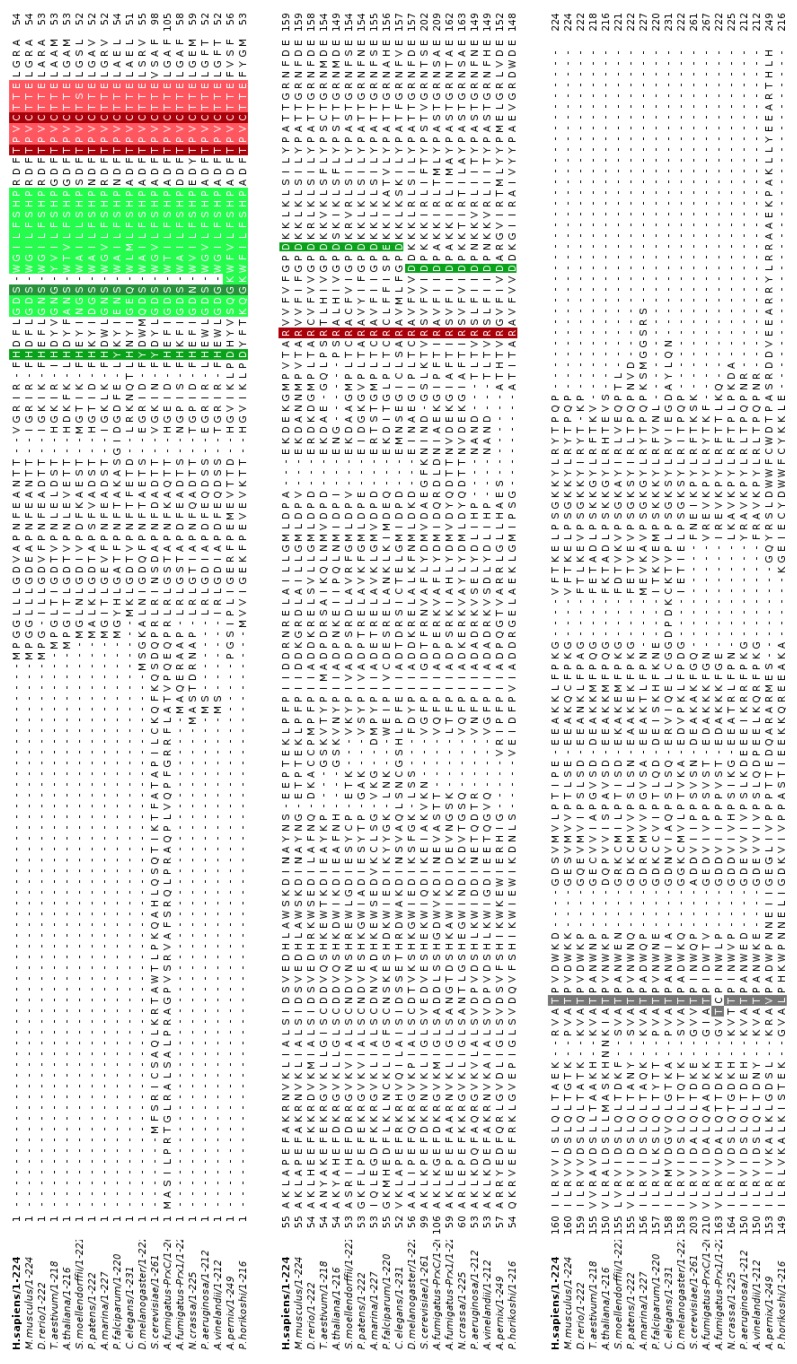
Alignment of representative Peroxiredoxins class 6 (Prdx6) enzymes from organisms that belong to diverse phylogenetic groups. Lysosomal putative target sequence (light green), phospholipase A_2_ (PLA_2_) catalytic triad (dark green), Prdx6 peroxidase motif (light red), peroxidatic catalytic triad (dark red) and the Thr that is putatively subjected to phosphorylation (gray) are highlighted.

Comparing the sequences of Prdx6 from different organisms, we observed that the peroxidase motif (PVCTTE) (Figure 1, light red) and the corresponding catalytic triad (Figure 1, dark red) are fully conserved, with the exception of one single replacement of amino acid with similar physico-chemical properties in Prdx6 from *Selaginella moellendorffii* (PVCTSE).

In contrast, the PLA_2_ motif (Figure 1) is not as conserved among Prdx6 enzymes as the peroxidase motif. The conserved His (His26 in human Prdx6) is present in most sequences (Figure 1, dark green), with few exceptions: a change to Tyr in *Plasmodium falciparum*, *Drosophila melanogaster* and *Saccharomyces cerevisiae* sequences or a change to Asp in the archaeal proteins from *Aeropyrum pernix* and *Pyrococcus horikoshii* (Figure 1, dark green). Likewise, Ser (Ser32 in human Prdx6) is conserved in many sequences, presenting a change to Gly in *Triticum aestivum*, *Azotobacter vinelandii* and the archaeal proteins, or to Gln or Asn in *Caenorhabditis elegans* and *Neurospora crassa*, respectively.

Finally, the last residue of the PLA_2_ triad, Asp (Asp140 in human Prdx6) is present in almost all sequences, with the exception of *P. falciparum* Prdx6, in which a Glu appears, maintaining the acidic properties. Notably, this conserved residue Asp may be located at two slightly distinct positions: in archaea, bacteria and fungus taxa, it is two amino acids backwards compared to other sequences (Figure 1, dark green). Possibly, this conserved Asp occupies similar position in the tertiary structure among distinct Prdx6 enzymes, keeping its function in catalysis.

The Thr residue (Thr177 in human Prdx6) that is subjected to phosphorylation in mammalian enzymes is highly conserved in almost all analyzed sequences. The exceptions are the archaeal ones (Figure 1, gray). It is worth mentioning that the Thr from AfPrx1 is one position backward compared to the other sequences. Although this Thr residue is highly conserved, the surrounding amino acid sequences are quite distinct among non-mammalian Prdx6 enzymes, especially in AfPrx1 and AfPrxC.

**Figure 2:**
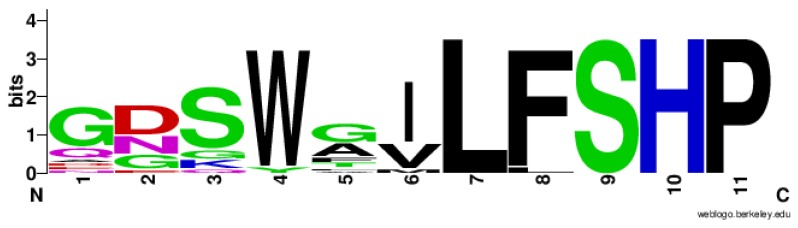
Graphical representation of residue conservation in the putative lysosomal target sequence. The letter size represents the conservation of each residue, while their color represents the chemical property of the corresponding amino acid. Polar amino acids Gly (G), Ser (S), Thr (T), Tyr (Y), Cys (C) are depicted in green; neutral amino acids Gln (Q) and Asn (N) are in magenta; basic amino acids Leu (L), Arg (R), His (H) are in blue; acidic amino acids Asp (D) and Glu (E) are in red; hydrophobic amino acids Ala (A), Val (V), Leu (L), Ile (I), Pro (P), Trp (W), Phe (F), Met (M) are in black. The logo was generated with the WebLogo application (http://weblogo.berkeley.edu).

In contrast, the lysosomal target sequence exhibits higher variability among Prdx6 enzymes (Figure 1, light green). Notably, the last 5 residues are highly conserved among the analyzed sequences (Leu-Phe/Leu-Ser-His-Pro) (LF/LSHP) (Figure 2). Considering the structural characteristics indicated above, we decided to investigate if other Prdx6 enzymes from non-mammalian organisms display PLA_2_ activity.

### 3.2. Structural Conservation of Prdx6 Motifs in TaPER1, AtPER1, AfPrx1, AfPrxC, and PaLsfA

**Figure 3:**
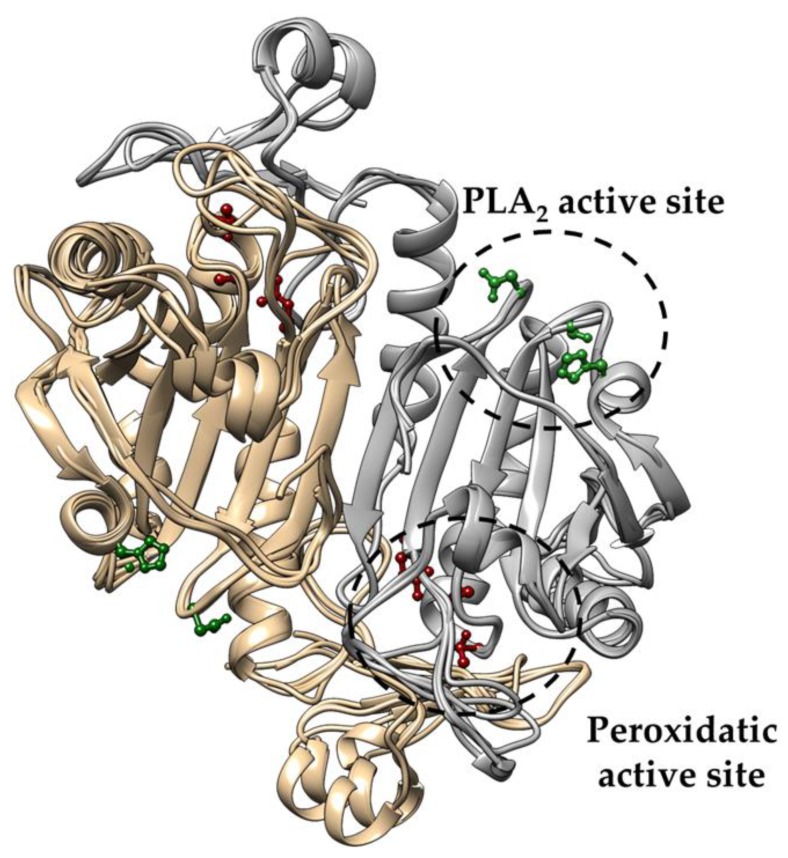
Superposition of the predicted structures of Prdx6 from *T. aestivum*, *A. thaliana*, *P. aeruginosa, A. fumigatus* (AfPrx1 and AfPrxC). One monomer is represented in copper and the other in gray in the antiparallel dimers. The PLA_2_ catalytic triad is depicted in green, and the peroxidatic catalytic triad in red. The predicted structures were generated using human Prdx6 as modeled by the SWISS-MODEL tool (https://swissmodel.expasy.org/).

**Figure 4:**
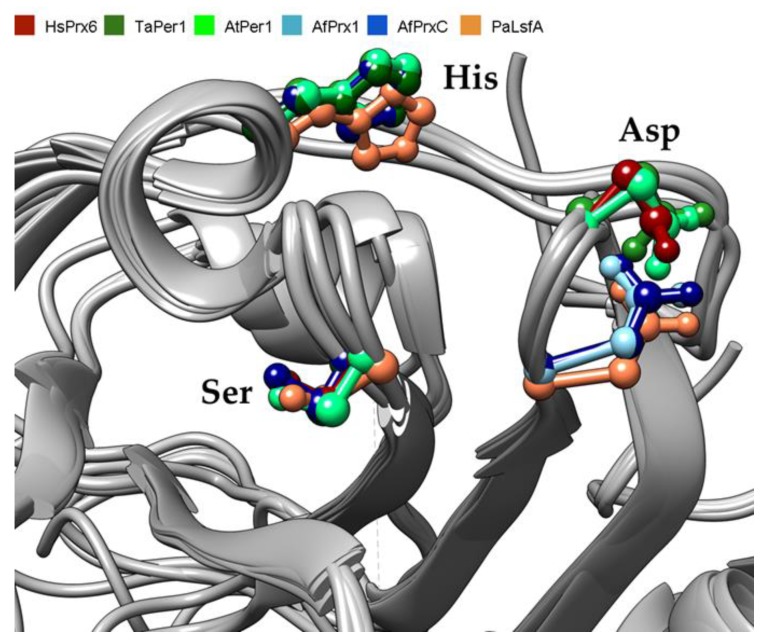
Close view of the PLA_2_ catalytic triad of six 1-Cys Prdxs from *H. sapiens* (red), *T. aestivum* (dark green), *A. thaliana* (light green), *A. fumigatus* (Prx1 in light blue and PrxC in dark blue), and *P. aeruginosa* (orange) superimposed.

Next, we investigated the three-dimensional conservation of the PLA_2_ catalytic triad by modeling the structures of TaPER1, AtPER1, AfPrx1, AfPrxC, and PaLsfA. The PLA_2_ and the peroxidatic active sites are conserved and are located at opposite sides of their thioredoxin fold (Figure 3), as previously described for mammalian Prdx6 [34]. Apparently, in all the predicted structures, the putative substrate (a phospholipid) for the PLA_2_ activity would fit in the corresponding cavity (Figure 3). Remarkably, the two distinct positions occupied by the conserved Asp residues in the amino acid sequences are reflected in the predicted three-dimensional structures: (1) the plant orthologues and the human Prdx6 have the Asp in a similar position, whereas (2) the orthologues from fungi and bacteria have the conserved Asp in a backward position (Figure 4). The main difference between non-mammalian Prdx6 and human orthologues is the Ser -->Gly substitution observed in TaPER1 (Figure 4). The structural similarities among these proteins suggest that they can display PLA_2_ activity.

### 3.3. Acidic, Ca^2+^-Independent PLA_2_ (aiPLA_2_) Activity of Non-Mammalian Prdx6

Next, we evaluated the PLA_2_ activity of four (4) non-mammalian enzymes. All of the analyzed proteins displayed PLA_2_ activity at acidic conditions (pH 4) in a Ca^2+^-independent manner (Table 1), consistent with the so-called acidic Ca^2^^+^-independent phospholipase A_2_ (aiPLA_2_) described before for the human Prdx6 [34]. In addition, these activities attained similar values, ranging from 3.38 for PaLsfA to 6.09 for AfPrx1 nmol/min/mg protein (Table 1).

### 3.4. Phosphorylation at the C-terminal Thr Is Also Conserved Among Non-Mammalian Prdx6

Thr177 phosphorylation of human Prdx6 increases its PLA_2_ activity, especially at neutral pH [15]. Therefore, we investigated if human Erk2 could phosphorylate homologous Thr residues in non-mammalian Prdx6 proteins. Several phosphorylation sites were found in the two plant proteins (TaPER1 and AtPER1) and also in the mitochondrial Prdx6 from *A. fumigatus* (AfPrxC) (data not shown), including the Thr homologous of human Prdx6 Thr177. However, only TaPER1 presented an elevated score (Figure S1). In contrast, AfPrx1 and PaLsfA proteins were not phosphorylated as no phosphorylated peptide was found for either protein by mass spectrometry (Table 2).

**Figure 5:**
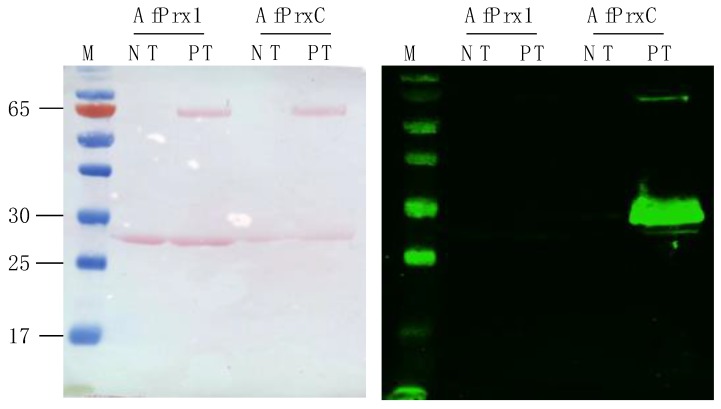
Evaluation of AfPrx1 and AfPrxC phosphorylation. Left panel represents the membrane stained with Ponceau S as the loading control. Right panel represents the Western Blot using the primary antibody for phosphorylated MmPrdx6. Lane 1: molecular ladder, the numbers at the left side represent the molecular weight (kDa); lane 2: non-treated (NT) AfPrx1; lane 3: AfPrx1 after phosphorylation treatment (PT); lane 4: non-treated AfPrxC; lane 5: AfPrxC after phosphorylation treatment. The lower bands represent the Prdx and upper bands represent the kinase Erk2. Phosphorylation treatment is described in the Material and Methods section.

We also verified the possible Thr phosphorylation of AfPrx1 and AfPrxC by western blot using an antibody developed to detect phosphorylated Thr residues in mouse Prdx6 [17]. A band for phosphorylated protein was only observed for AfPrxC (Figure 5), similar to the mass spectrometry results (Table 2).

### 3.5. Influence of Phosphorylation and Inhibition with MJ33 on PLA_2_ Activity of AfPrx1 and AfPrxC

Then, the effects of the phosphorylation treatment on the PLA_2_ activity were determined for AfPrx1 and AfPrxC. Both proteins had their activities increased almost four (4) times after treatment with human Erk2 (Table 3). Next, we evaluated if MJ33, a classical competitive inhibitor of mammalian Prdx6, could inhibit the fungal Prdxs [7]. MJ33 inhibited about 85% of the aiPLA_2_ activities of AfPrx1 and AfPrxC.

## 4. Discussion

The aiPLA_2_ activity of mammalian Prdx6 was first detected in 1992 in isolated rat lungs [35] and then in epithelial cell cultures [36]. The protein was identified, cloned and enzymatically characterized as a lysosomal type, Ca^2^^+^-independent PLA_2_ [7]. Shortly after that, the Cys-based peroxidase activity of human Prdx6 was described. Then, it was realized that these aiPLA_2_ and the human Prdx6 enzymes shared the same amino acid sequence [7]. Therefore, Prdx6 is a bi-functional enzyme with Cys-based peroxidase and aiPLA_2_ activities. Later, the biological relevance of this aiPLA_2_ activity was demonstrated, that is, phospholipid turnover, repair of cell membranes;, and NADPH oxidase 2 (NOX2) activation [16,17,37].

Intriguingly, aiPLA_2_ activity had not yet been evaluated in non-mammalian Prdx6 proteins before this work. Here, we observed that five non-mammalian Prdx6 displayed conserved amino acid sequenced and structural features (Figure 1, Figure 2, Figure 3 and Figure 4) compatible with enzymes endowed with aiPLA_2_ activities. Indeed, the overall structures modeled here were very similar to the human Prdx6. In all cases, accessible active sites were present and the conserved catalytic triads were in the expected position. In mammalian Prdx6, it is proposed that the peroxidized *sn*-2 acyl phospholipid chain enters into the pocket, where the peroxidatic Cys is located on the bottom, and the PLA_2_ active site is on the top. Therefore, Prdx6 can reduce and hydrolyze the peroxidized phospholipid [38]. Next, we will consider how each specific featured related to PLA_2_ activity is conserved among non-mammalian Prdx6 enzymes.

In human Prdx6, His26 is postulated to play roles in phospholipid binding, as the positive charge of this residue can interact with the negative charge of the substrate [12]. In some Prdx6 sequences, a Tyr residue replaces this conserved His (proteins from *P. falciparum*, *D. melanogaster* and *S. cerevisiae*). We did not investigate any of these enzymes here.

Ser32 of human Prdx6 is also important for the interaction of the enzyme with the substrate and for stabilization of protein structure [12]. Almost 70% of the analyzed sequences possess this residue conserved. However, four homologs, including the TaPER1, possess Gly in the same position. Nevertheless, TaPER1 displayed PLA_2_ activity at similar levels to the other Prdx6 analyzed in the present work. Notably, the mutation of catalytic Ser to Asn, Thr, Ala or Asp in human lipoprotein lipase (LPL) abolished the enzymatic activity but when this same Ser residue was mutated to Gly, the activity was maintained [39].

Asp140 of human Prdx6 plays a central role in PLA_2_ catalysis [12] and is the most conserved residue of the catalytic triad. Indeed, the conserved Asp is replaced in only one case (from *P. falciparum*) by Glu, which is also a negatively charged residue. Notably, the conserved Asp residue can be located at two distinct positions. The proteins analyzed here possess this conserved Asp residue in either one of the two positions (Figure 1; Figure 4) and all of them displayed similar PLA_2_ activity (Table 1). Therefore, it is reasonable to assume that these differences in the Asp positioning do not interfere with PLA_2_ activity.

Thr177 of recombinant human Prdx6 when phosphorylated caused a 30-fold increase in PLA_2_ activity [40] and is conserved in all sequences, excluding the archaeal proteins. Therefore, we investigated if non-mammalian Prdx6 enzymes could be phosphorylated by human Erk2. Three (TaPER1, AtPER1, AfPrxC) out of the five non-mammalian Prdx6 were phosphorylated at the conserved Thr residue (Table 2). Possibly, this conserved Thr of TaPER1, AtPER1, AfPrxC is phosphorylated by plant and fungal kinases, which could increase their PLA_2_ activity. Indeed, AfPrxC treated with human Erk2 had a four-fold increase in its activity (Table 3). Although, we have no experimental evidence that PaLsfA and AfPrx1 are phosphorylated, we cannot exclude the possibility that these peroxidases undergo this post-translational modification in vivo. It is possible that native kinases in *P. aeruginosa* and *A. fumigatus* might phosphorylate these Prdx6 enzymes.

The investigation of the physiological meaning of PLA_2_ activity in Prdx6 enzymes is complex as these proteins also display peroxidase activity. Indeed, participation of PLA_2_ activities of mammalian Prdx6 in distinct cellular processes were revealed several years after they were first characterized. For instance, the human alveoli are covered by surfactant, a surface active material able to reduce surface tension at the alveolar air-liquid interface [41]. This function prevents the alveoli from collapsing at end-respiration [42]. Pulmonary surfactant is composed of approximately 90% lipids and 10% protein and the major component of surfactant is the amphiphatic phospholipid DPPC (around 40% total) [43]. This layer is subjected to constant remodeling and the mammalian Prdx6 are important in this process of recycling in lamelar bodies (acid organelles) [13,16,17]. Since *A. fumigatus* and *P. aeruginosa* colonize the lungs, starting from alveoli, it is reasonable to speculate that the PLA_2_ activity of these proteins (AfPrx1, AfPrxC and PaLsfA) might contribute to the colonization process favoring the lung tissue invasion and colonization.

Accordingly, several phospholipases produced by pathogenic bacteria and fungus are virulence factors [44,45]. In *P. aeruginosa*, for example, ExoU is injected directly into the cytoplasm of host cells through the type III secretion system [46] and rapidly destroys the cell membranes of mammalian cells by its PLA_2_ activity [47]. The presence of ExoU is associated with antibiotic resistance and the severe outcome of many infections [48].

During the infection of mammalian hosts, phospholipase enzymes released by fungi can play important roles in tissue invasion and nutrient acquisition [49,50]. In *A. fumigatus*, two phospholipase B (*plb*) genes (*Afplb1* and *Afplb2*) encode two secreted PLB and are upregulated in the presence of the major component of lung surfactant (DPPC) [44,50], substrate analyzed as substrate in the present work. Interestingly, the AfPrx1 was detected in the secretoma of *A. fumigatus* [51] and can cleave DPPC in the *sn*-2 position (present work).

In plants, PLAs are involved in a wide range of cellular processes, including pollen and seed development, protection against water loss and also during jasmonic acid production, a compound related to signaling during plant defenses to distinct stressful conditions [51]. Interesting, HvPER1 (from *Hordeum vulgares*) and AtPER1 are highly expressed and accumulated in nucleus from embryos, especially during the final step of seed development, the desiccation step [22,52]. At this stage, the elevated production of reactive oxygen species is associated to the extreme loss of water along with the reduction in metabolism and antioxidant enzymatic defenses [53,54].

## 5. Conclusions

This is the first description of PLA_2_ activities for non-mammalian Prdx6 enzymes. Notably, the activity of the mammalian Prdx6 (≈ 100 nmol/min/mg protein) is about 20 times the activity obtained for non-mammalian Prdx6 (≈ 5 nmol/min/mg protein). Possibly, the liposomes used herein were not optimal substrates for non-mammalian Prdx6, as these lipids were designed to mimic human pulmonary surfactants. Indeed, the investigation of the PLA_2_ activities for the Prdx6 enzymes represents an open field for future research as the roles of lipids in cellular signaling is increasingly recognized.

## Figures and Tables

**Table 1:** PLA_2_ specific activity (nmol/min/mg protein) for non-mammalian Prdx6 at different pHs.

Organism	Protein	Activity (nmol/min/mg prot.)	
		pH 4	pH 7
*T. aestivum*	TaPER1	4.46 ± 0.1	0.06 ± 0.01
*A. fumigatus*	AfPrx1	6.09 ± 0.1	0.7 ± 0.2
	AfPrxC	4.91 ± 0.2	0.5 ± 0.1
*P. aeruginosa*	PaLsfA	3.38 ± 0.1	0.04 ± 0.002

**Table 2:** MS/MS analysis of 1-Cys Prdx purified proteins after human Erk2 treatment and digestion. Only the peptides containing the Thr homologous to Thr177 of human Prdx6 are shown. The presence of phosphorylation is indicated in red (+Phospho).

Organism	Protein	Fragment Detected after Digestion	Observed Mass	Charge	Score	Error (ppm)
*T. aestivum*	TaPER1	HKVAT(+Phospho)PANWNPGECVVIAPGVSDDEAKK	768.11	4	61	5.53
		HKVATPANWNPGECVVIAPGVSDDEAKK	748.12	4	85	4.89
*A. thaliana*	AtPER1	ALDSLLMASKHNNKIAT(+Phospho)PVNWKPDQPVVISPAVSDEEAK	859.43	5	31	5.77
		ALDSLLMASKHNNKIATPVNWKPDQPVVISPAVSDEEAK	843.44	5	29	2.38
*A. fumigatus*	AfPrx1	VVDALQTTDKHGVTCPINWLPGDDVIIPPPVSTEDAK	1000.25	4	42	5.96
		VVDALQTTDKHGVTCPINWLPGDDVIIPPPVSTEDAK	1000.26	4	50	2.06
	AfPrxC	VIDALQAADKKGIAT(+Phospho)PIDWTVGEDVIVPPSVSTEDAK	983.00	4	19	0.31
		VIDALQAADKKGIATPIDWTVGEDVIVPPSVSTEDAK	963.01	4	56	2.35
*P. aeruginosa*	PaLsfA	VIDSLQLTDEHKVATPANWEDGDEVVIVPSLKDEEEIKR	1473.07	3	111	8.59
		VIDSLQLTDEHKVATPANWEDGDEVVIVPSLKDEEEIKR	1473.07	3	65	7.64

**Table 3:** PLA_2_ specific activity (nmol/min/mg protein) for non-mammalian Prdx6 in different pH, phosphorylation treatment with Erk2 and after inhibition with 1-hexadecyl-3-trifluoro-ethylglycero-s*n*-2-phosphomethanol (MJ33). ND: non-determined.

Treatment	AfPrx1		AfPrxC	
	pH 4	pH 7	pH 4	pH 7
No treatment	6.09 ± 0.1	0.7 ± 0.2	4.91 ± 0.2	0.5 ± 0.1
Erk2	21.85 ± 0.5	20.96 ± 0.3	18.69 ± 0.2	18.47 ± 0.1
MJ33	0.85 ± 0.05	ND	0.89 ± 0.01	ND

## Supplementary Materials

The following are available online at https://www.mdpi.com/2076-3921/8/3/52/s1, Figure S1: MS/MS fragmentation profile for the non-mammalian Prdx6. The results were obtained using MASCOT 2.4 software (Matrix Science Ltd., London, United Kingdom, Redoxoma-FAPESP user license 10.10.1.46/Mascot).
