# Supplementary material for: Non-Mammalian Prdx6 Enzymes (Proteins with 1-Cys Prdx Mechanism) Display PLA2 Activity Similar to the Human Orthologue
Source: Antioxidants (Basel). 2019 Mar 1;8(3):52. doi: 10.3390/antiox8030052 (PMC6466579; doi:10.3390/antiox8030052)
Supplement: Supplementary file 1 [file antioxidants-08-00052-s001.pdf]

TaPER1

Not treated

Peptide detected

MS/MS Fragmentation of **HKVATPANWNPGECVVIAPGVSDDEAKK**
Found in **Q6W8Q2** in **t_aestivum**, 1-Cys peroxiredoxin PER1 OS=Triticum aestivum OX=4565 GN=PER1 PE=2 SV=1

MS/MS spectra

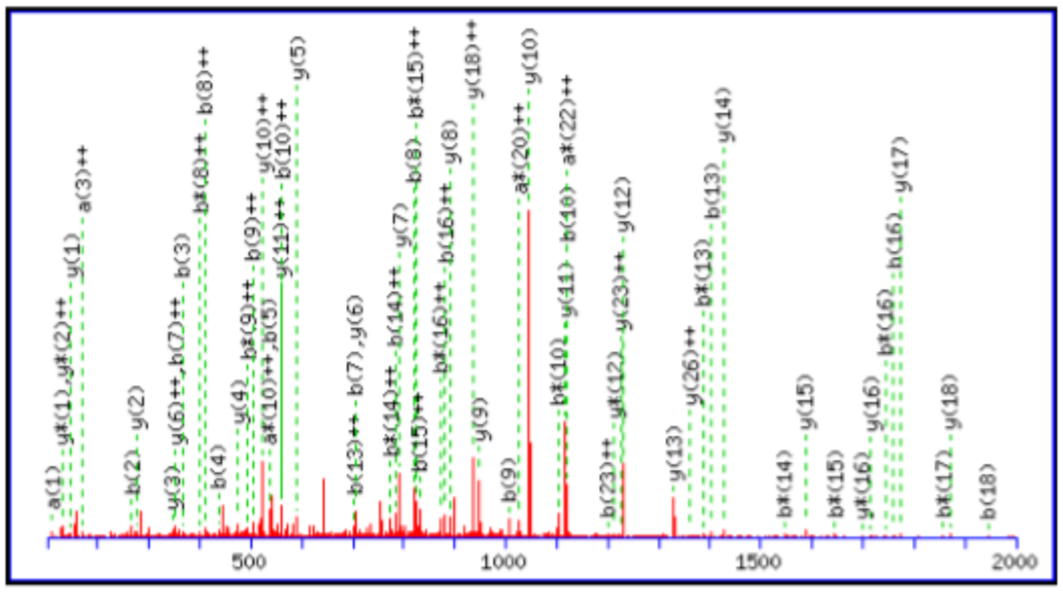


```
Monoisotopic mass of neutral peptide Mr(calc): 2988.4763
Fixed modifications: Carbamidomethyl (C) (apply to specified residues or termini only)
Ions Score: 85  Expect: 4.8e-007
Matches : 64/320 fragment ions using 143 most intense peaks   (help)
```

After phosphorylation assay

Peptide detected

MS/MS Fragmentation of **HKVATPANWNPGECVVIAPGVSDDEAKK**
Found in **Q6W8Q2** in **t_aestivum**, 1-Cys peroxiredoxin PER1 OS=Triticum aestivum OX=4565 GN=PER1 PE=2 SV=1

MS/MS spectra

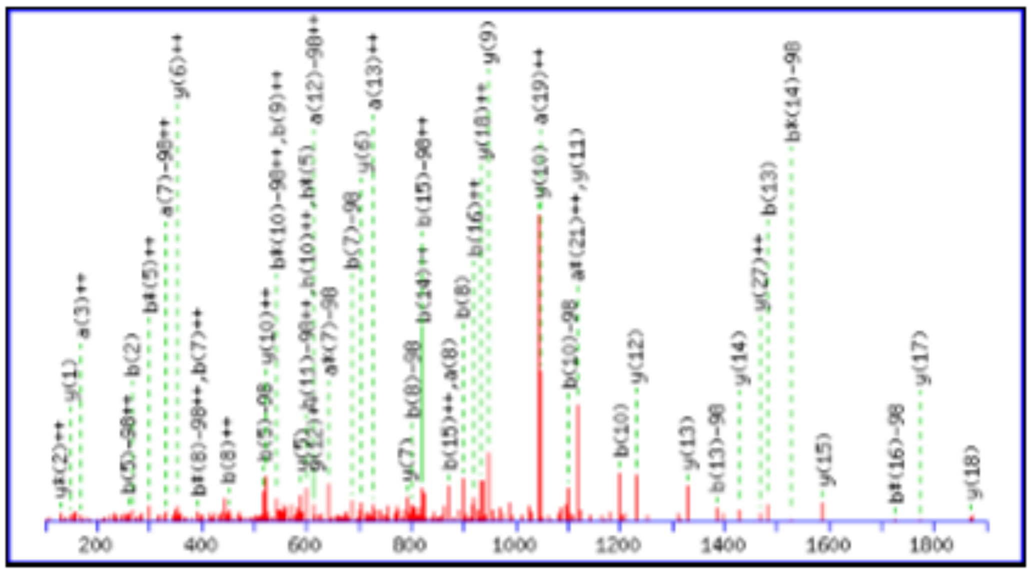


```
Monoisotopic mass of neutral peptide Mr(calc): 3068.4427
Fixed modifications: Carbamidomethyl (C) (apply to specified residues or termini only)
Variable modifications:
T5     : Phospho (ST), with neutral losses 0.0000(shown in table), 97.9769
Ions Score: 61  Expect: 0.00013
Matches : 52/520 fragment ions using 76 most intense peaks   (help)
```

AtPER1

Not treated

Peptide detected

MS/MS Fragmentation of **ALDSLLMASKHNNKIATPVNWKPDQPVVISPAVSDEEAK**
Found in **A0A178W968** in **a_thaliana**, PER1 OS=Arabidopsis thaliana OX=3702 GN=AXX17_At1g42190 PE=4 SV=1

MS/MS spectra

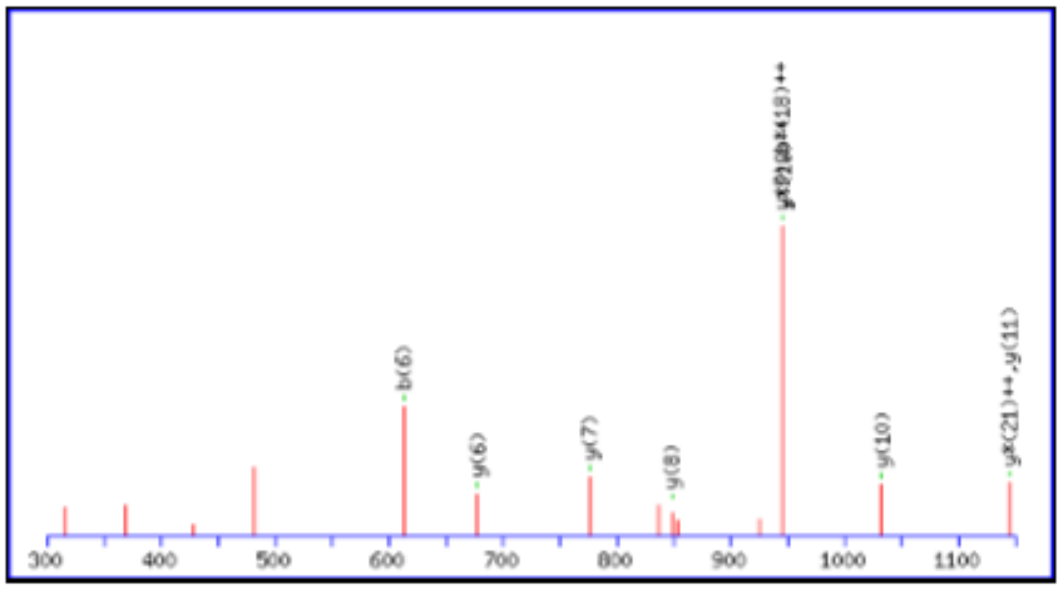


```
Monoisotopic mass of neutral peptide Mr(calc): 4212.1888
Fixed modifications: Carbamidomethyl (C) (apply to specified residues or termini only)
Ions Score: 29  Expect: 0.84
Matches : 10/420 fragment ions using 14 most intense peaks   (help)
```

After phosphorylation assay

Peptide detected

MS/MS Fragmentation of **ALDSLLMASKHNNKIATPVNWKPDQPVVISPAVSDEEAK**
Found in **A0A178W968** in **a_thaliana**, PER1 OS=Arabidopsis thaliana OX=3702 GN=AXX17_At1g42190 PE=4 SV=1

MS/MS spectra

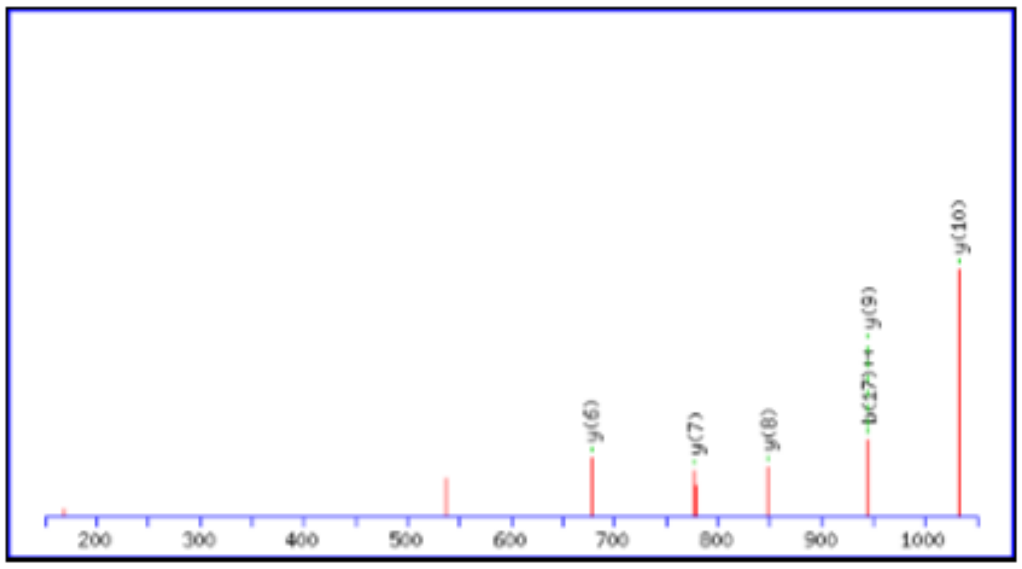


```
Monoisotopic mass of neutral peptide Mr(calc): 4292.1552
Fixed modifications: Carbamidomethyl (C) (apply to specified residues or termini only)
Variable modifications:
T17     : Phospho (ST), with neutral losses 0.0000(shown in table), 97.9769
Ions Score: 31  Expect: 0.78
Matches : 6/660 fragment ions using 9 most intense peaks   (help)
```

AfPrx1

Not treated

Peptide detected

MS/MS Fragmentation of **VVDALQTTDKHGVTCPINWLPGDDVIIPPPVSTEDAK**
Found in **A0A0J5PDS6** in **a_fumegatus**, Antioxidant protein LsfA OS=Aspergillus fumigatus Z5 OX=1437362 GN=Y699_06242 PE=4 SV=1

MS/MS spectra

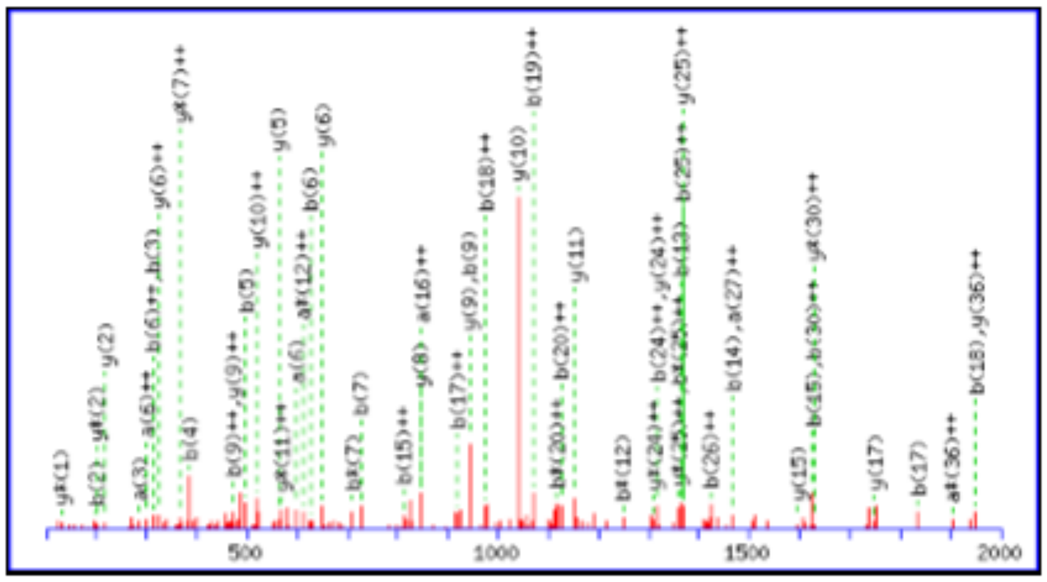


```
Monoisotopic mass of neutral peptide Mr(calc): 3997.0143
Fixed modifications: Carbamidomethyl (C) (apply to specified residues or termini only)
Ions Score: 50  Expect: 0.0068
Matches : 56/412 fragment ions using 116 most intense peaks   (help)
```

After phosphorylation assay

Peptide detected

MS/MS Fragmentation of **VVDALQTTDKHGVTCPINWLPGDDVIIPPPVSTEDAK**
Found in **A0A0J5PDS6** in **a_fumegatus**, Antioxidant protein LsfA OS=Aspergillus fumigatus Z5 OX=1437362 GN=Y699_06242 PE=4 SV=1

MS/MS spectra

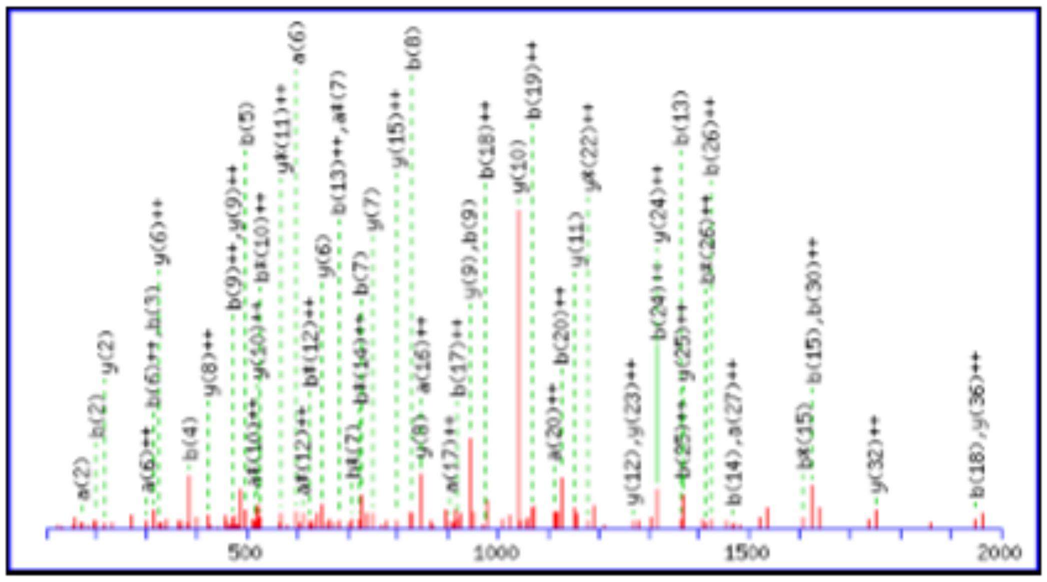


```
Monoisotopic mass of neutral peptide Mr(calc): 3997.0143
Fixed modifications: Carbamidomethyl (C) (apply to specified residues or termini only)
Ions Score: 42  Expect: 0.048
Matches : 58/412 fragment ions using 140 most intense peaks   (help)
```

AfPrxC

Not treated

Peptide detected

MS/MS Fragmentation of **VIDALQAADKKGIATPIDWTVGEDVIVPPSVSTEDAK**
Found in **A0A0J5PIP5** in **a_fumegatus**, AhpC/TSA family thioredoxin peroxidase OS=Aspergillus fumigatus Z5 OX=1437362 GN=Y699_05401 PE=4 SV=1

MS/MS spectra

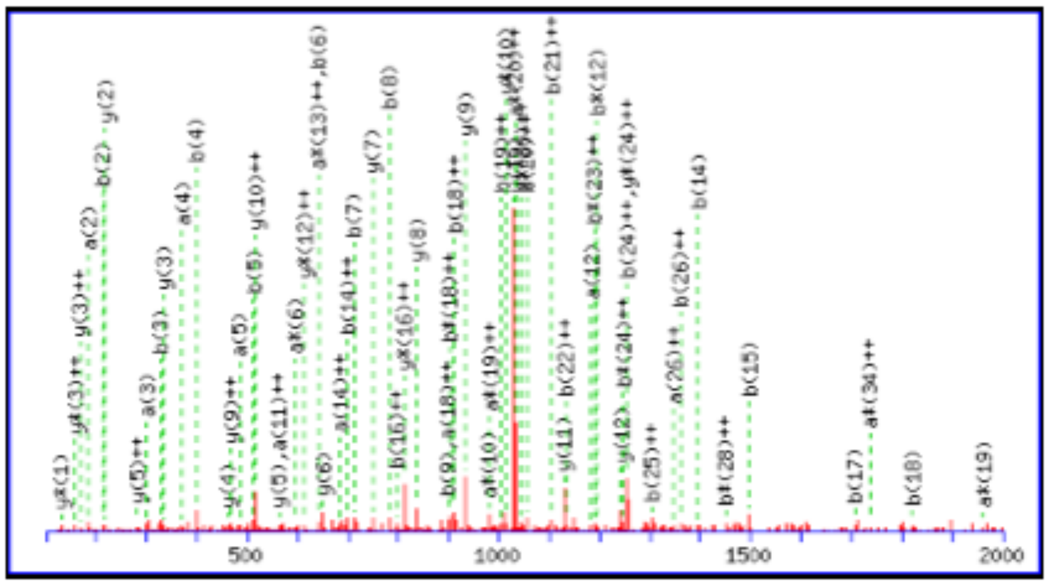

Monoisotopic mass of neutral peptide Mr(calc): 3848.0095
Fixed modifications: Carbamidomethyl (C) (apply to specified residues or termini only)
Ions Score: 56 Expect: 0.0012
Matches : 65/412 fragment ions using 170 most intense peaks (help)

After phosphorylation assay

Peptide detected

MS/MS Fragmentation of **VIDALQAADKKGIATPIDWTVGEDVIVPPSVSTEDAK**
Found in **A0A0J5PIP5** in **a_fumegatus**, AhpC/TSA family thioredoxin peroxidase OS=Aspergillus fumigatus Z5 OX=1437362 GN=Y699_05401 PE=4 SV=1

MS/MS spectra

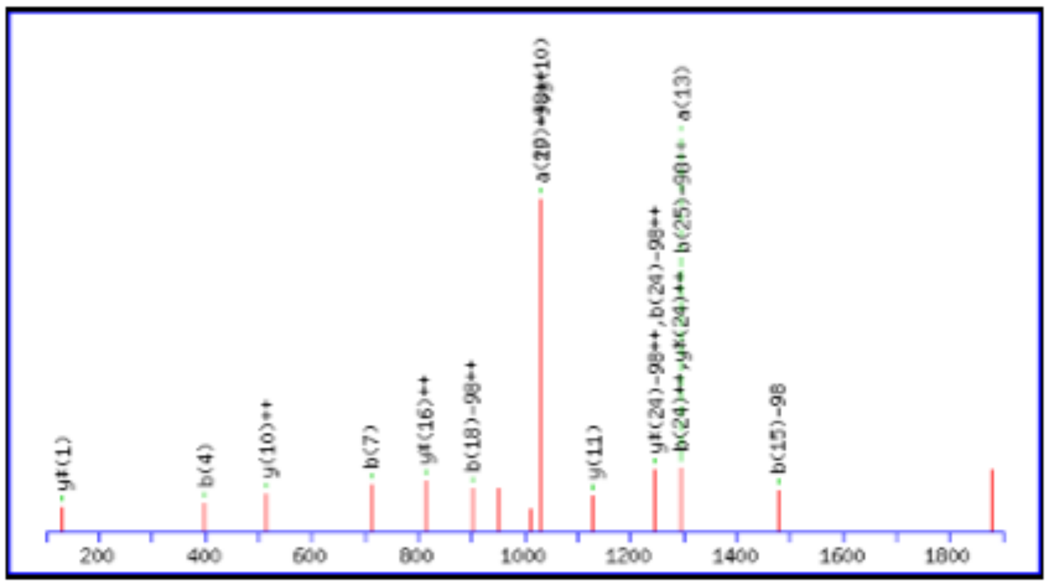

Monoisotopic mass of neutral peptide Mr(calc): 3927.9758
Fixed modifications: Carbamidomethyl (C) (apply to specified residues or termini only)
Variable modifications:
T15   : Phospho (ST), with neutral losses 97.9769(shown in table), 0.0000
Ions Score: 19 Expect: 8.6
Matches : 17/644 fragment ions using 17 most intense peaks (help)

PaLsfA

Not treated

Peptide detected

MS/MS Fragmentation of **VIDSLQLTDEHKVATPANWEDGDEVVIVPSLKDEEEIKR**
Found in **A0A071KYB2** in **p_aeruginiosa**, Peroxidase OS=Pseudomonas aeruginosa OX=287 GN=tsaA_2 PE=4 SV=1

MS/MS spectra

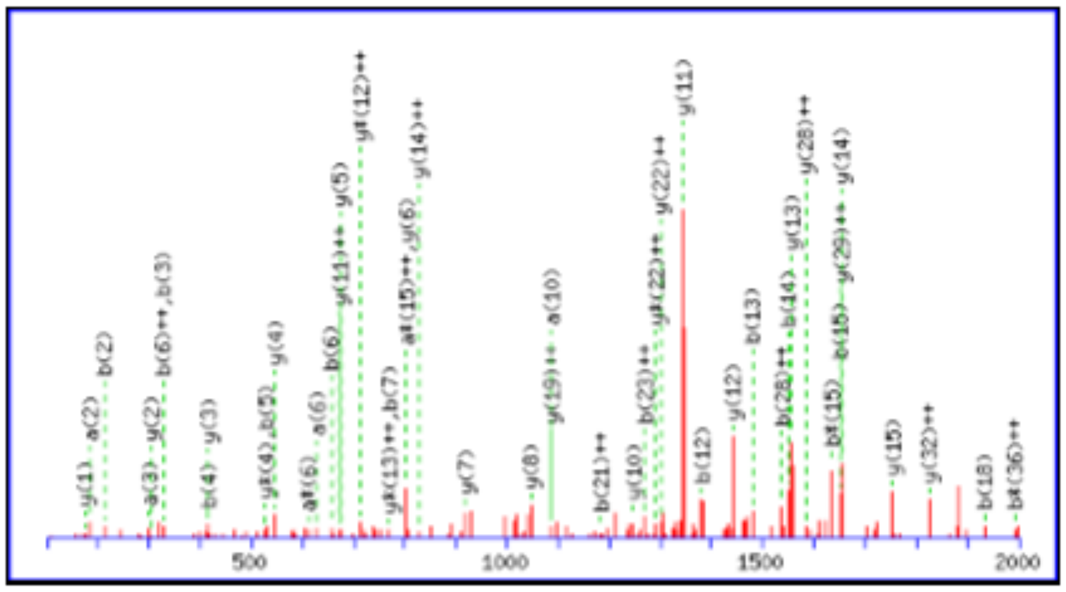

Monoisotopic mass of neutral peptide Mr(calc): 4416.2336
Fixed modifications: Carbamidomethyl (C) (apply to specified residues or termini only)
Ions Score: 65 **Expect:** 0.00022
Matches : 48/436 fragment ions using 123 most intense peaks (help)

After phosphorylation assay

Peptide detected

MS/MS Fragmentation of **VIDSLQLTDEHKVATPANWEDGDEVVIVPSLKDEEEIKR**
Found in **A0A071KYB2** in **p_aeruginiosa**, Peroxidase OS=Pseudomonas aeruginosa OX=287 GN=tsaA_2 PE=4 SV=1

MS/MS spectra

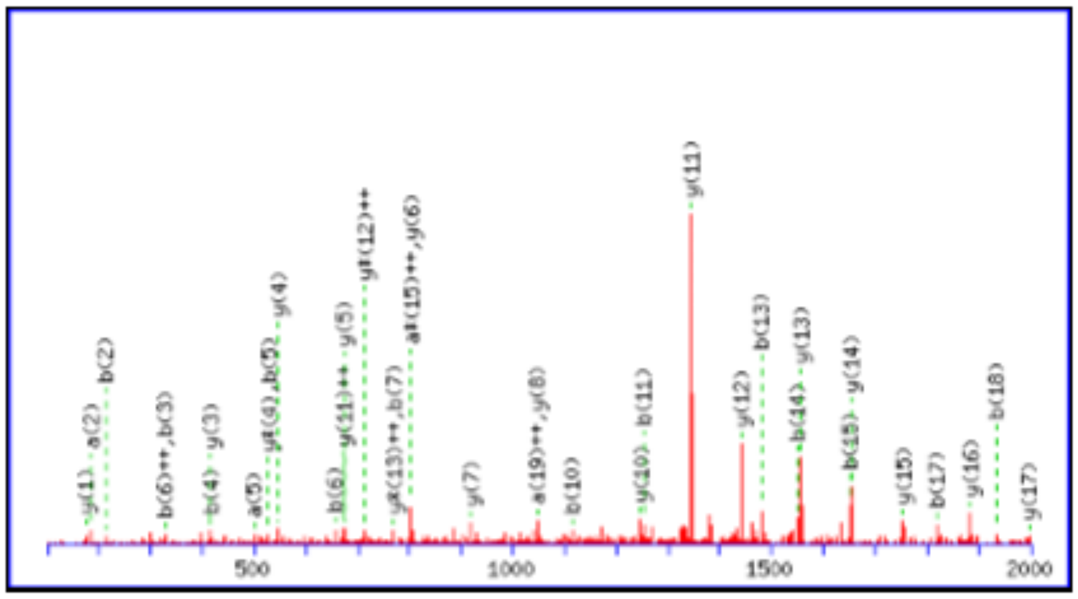

Monoisotopic mass of neutral peptide Mr(calc): 4416.2336
Fixed modifications: Carbamidomethyl (C) (apply to specified residues or termini only)
Ions Score: 111 **Expect:** 6.1e-009
Matches : 37/436 fragment ions using 57 most intense peaks (help)
